# Effect of UV-C Irradiation, Storage and Subsequent Cooking on Chemical Constituents of Fresh-Cut Potatoes

**DOI:** 10.3390/foods10081698

**Published:** 2021-07-22

**Authors:** Zdenka Pelaić, Zrinka Čošić, Sandra Pedisić, Maja Repajić, Zoran Zorić, Branka Levaj

**Affiliations:** Faculty of Food Technology and Biotechnology, University of Zagreb, Pierottijeva 6, 10000 Zagreb, Croatia; zcosic@pbf.hr (Z.Č.); spedisic@pbf.hr (S.P.); maja.repajic@pbf.unizg.hr (M.R.); zzoric@pbf.hr (Z.Z.); blevaj@pbf.hr (B.L.)

**Keywords:** UV-C, fresh-cut potatoes, storage, cooking, phenolics, sugars, acrylamide

## Abstract

UV-C irradiation successfully reduces the growth of microorganisms, but it can also affect the content of phenolics and sugars of fresh-cut potatoes (FCP). This could consequently alter antioxidant capacity of FCP or its potential for acrylamide formation. Therefore, this paper investigates the influence of UV-C irradiation on the content of phenolics [chlorogenic acid (CA)] and individual sugars during storage of FCP as well as after cooking. Acrylamide was also monitored in FCP after frying. Potato slices pre-treated with sodium ascorbate solution and vacuum-packaged were UV-C irradiated for 0, 3, 5, and 10 min in order to obtain irradiation doses of 0, 1.62, 2.70, and 5.40 kJ m^−2^, respectively, stored for 23 days (+6 °C), and subsequently boiled and fried. As the applied dose and storage duration increased, the CA content in raw FCP decreased (it retained for 75.53–88.34%), while the content of sugars as well as acrylamide in fried FCP increased. Although the increase was the most noticeable at the applied dose of 2.70 kJ m^−2^, the acrylamide content was always below proposed limit. Boiling and frying reduced the content of CA and sugars. In spite of certain alterations, applied doses of irradiation can ensure acceptable product in regard to phenolics and sugars, and acrylamide content particularly.

## 1. Introduction

Minimal processing (washing, peeling, cutting, etc.) of fruits and vegetables leads to tissue disruption and increased microbial growth, enzymatic activity, respiration rate, and ethylene production as well as other undesirable changes. All of these alterations cause quality deterioration and consequently a reduced shelf-life of fresh-cut products [1,2]. With the aim to prevent a microbiological spoilage, multiple methods, and techniques are being investigated, including non-ionizing UV-C technology at optimal wavelength of 254 nm. It has already been proven as an effective method in this regard and therefore can extend the shelf-life of fresh-cut products [3,4]. In accordance with this primary role of UV-C irradiation in food processing, it should preserve the quality of the product which greatly depends on the applied intensity of irradiation and exposure time [5]. Moreover, a number of other factors affect the effectiveness of irradiation, such as the properties of the plant material, the anti-browning agents or packaging material used [6,7]. The UV-C irradiation can inhibit enzyme activity and, consequently, reduce the browning of fresh-cut products [8]. Further, it can modify the flavor [9] or increase the content of phenolics and other bioactive compounds [9,10]. Increased content of phenolics may be, inter alia, a result of beneficial effect of UV-C, which stimulates the production of phenylalanine ammonia-lyase (PAL) [11]. In turn, this enzyme catalyzes the synthesis of phenolics what can consequently lead to an improved resistance to microorganisms. However, some of the authors observed a negative irradiation effect, such as induced browning, through the storage [12], breakages of cellular membranes [13], increased respiration rate [2], and decreased content of phenolics as well as antioxidant capacity through the storage, when higher UV-C irradiation was applied [14].

Regarding potatoes, the effect of tuber irradiation was mainly studied on weight loss, rot resistance, or the content of sugars during storage [15,16,17]. An alleviated accumulation of reducing sugars (fructose and glucose) was observed during low-temperature storage of irradiated tubers [15], which consequently can lead to a decreased ability of acrylamide formation. However, a recent study showed that UV-C irradiation of potato tubers two days before the preparation of semi-finished products can increase the acrylamide content in fried potatoes, although the content of sugars in tubers was not analyzed in this study [18]. Reducing sugars, along with free amino acid asparagine, are the precursors in Maillard’s reactions in which acrylamide is formed in potatoes fried at temperatures above 120 °C [19]. Acrylamide is a neurotoxic organic compound, probably cancerogenic to humans, as it is classified in group 2A by the International Agency for Research on Cancer (IARC) [20]. According to the EU Commission Regulation (2017/2158) [21], upper acrylamide limit in potato products is 750 µg kg^−1^ of fresh weight (FW). Once acrylamide is distributed throughout the organs, it is metabolized, inter alia, to glycidamide, the formation of which is considered to be the basis of the acrylamide genotoxicity and carcinogenicity (EFSA 2015) [22]. 

Currently, only few studies have addressed the effect of UV-C irradiation on fresh-cut potatoes (FCP), primarily investigating its effect on enzymes and the content of phenolics [7,23]. Phenolics are non-nutritional phytochemicals and present a significant group of compounds in potatoes, primarily due to their antioxidant properties. Besides, they are involved in enzymatic reactions of browning which alters the appearance and quality of products. PAL is wound-induced enzyme and its activity is enhanced due to minimally processing, by which, as already mentioned, the formation of phenolics increases [24]. Phenolics act then as substrates in oxidation reactions catalyzed by polyphenol oxidase (PPO) and peroxidase (POD) enzymes. PPO catalyzes the hydroxylation of monophenols in o-diphenols and the oxidation of o-diphenols into o-quinones of which dark pigment melanin is formed by non-enzymatic reactions [25,26]. According to previous research, UV-C irradiation can reduce PPO activity and increase the content of total phenolics in FCP [7]. Still, although reduced, PPO activity was significantly increased with the increased UV-C dose [7]. On the other hand, Xie et al. [23] did not observe significant differences in PPO activity until the 13th day of storage, after which a significant decrease compared to control trend was present till 19th day storage. 

The scientific data on the effect of UV-C irradiation on the chemical constituents of FCP are scarce and mainly on raw samples [7]. Therefore, the aim of this study was to examine the effect of several doses of UV-C irradiation (0, 1.62, 2.70, and 5.40 kJ m^−2^) on the content of phenolics and sugars in FCP as well as on the content of acrylamide formed in fried FCP. Additionally, this study also included the monitoring of these compounds influenced by FCP storage time and cooking method.

## 2. Material and Methods

### 2.1. Plant Material

Potato (*Solanum tuberosum* L.) tubers of cv. Birgit were used for the experiment. Tubers were harvested in the Croatian region of Slavonia during 2019 and prior storage were treated with an anti-sprouting agent (Gro Stop Basis and Gro Stop Fog, Certis Europe B.V., Cambridge, UK). Before the analysis, tubers were stored one month in the dark at 8 °C and relative humidity app. 100%.

### 2.2. Chemicals and Standards

Formic acid, *n*-hexane, acetonitrile (HPLC grade) and methanol (HPLC grade) were purchased from Sigma-Aldrich (Steinheim, Germany) as well as standards: acrylamide (>99%), chlorogenic acid, caffeic acid, *p*-coumaric acid, catechin, rutin, D-(−)-fructose (≥99% GC), D-(+)-glucose (≥99.5% GC), and D-(+)-sucrose (≥99.5% GC). The QueChERS salt packet (4 g MgSO_4_ and 0.5 g NaCl) and QueChERS d-SPE salts (150 mg MgSO_4_ and 50 mg PSA) were purchased from Agilent Technologies (Santa Clara, CA, USA). Water was of Milli-Q quality (Millipore Corp., Bedford, MA, USA).

### 2.3. Sample Preparation 

Sample preparation was conducted according to the procedure described by Dite Hunjek et al. [27]: only uniform and undamaged tubers were selected, washed, drained, and hand-peeled. Afterwards, tubers were sliced (0.4 cm) using a commercial slicer (SFS 1001 GR, Sencor, Ricany, Czech Republic) and dipped for 3 min into sodium ascorbate solution (2%, *w*/*v*) with a sample/solution (g mL^−1^) ratio of 1:4. Using SmartVac SV 750 (Status, Metlika, Slovenia) 4–6 drained slices were then vacuum packaged in one single layer within the polyamide/polyethylene double-layered (100 and 130 µm) vacuum bags (Status, Metlika, Slovenia).

### 2.4. UV-C Treatment

After vacuum packaging, potato slices were UV-C treated using an UV-C chamber (UVpro EKB 100, Orca GmbH, Kürten, Germany) equipped with four UV-C lamps (4xHNSL 24 W, maximal emission at 253.7 nm, UVpro) with two of them located 22 cm above and the other two under the perforated shelf. After 20 min of initial stabilization of the UV-C lamps, the samples (four bags) were placed on the certain place of the perforated shelf (previously tested) to ensure the uniformity of dose application. Doses of 0, 1.62, 2.70, and 5.40 kJ m^−2^ (UVCpro UVC-LOG radiometer, Orca GmbH, Kürten, Germany) were achieved by, 3 (3-UV-C), 5 (5-UV-C), and 10 min (10-UV-C) of irradiation and are expressed as a mean of 10-dose readings within the selected area. In order to compare UV-C treated and untreated FCP, control sample (control, 0) was also prepared by the same procedure described in Section 2.3 without further UV-C treatment. Samples were then stored at 6 ± 1 °C for 23 days. At the beginning of the storage (0) and on the 8th, 11th, 15th, and 23rd day of storage samples were cooked (boiled and fried), and raw samples as well as cooked ones were analyzed. 

### 2.5. Cooking Treatments

Boiled potato slices were prepared by boiling in water (water:sample = 5:1) at 100 °C for 15 min, while fried ones by frying in sunflower oil (oil:sample = 1.5 L:180 g) at initial temperature of 180 °C for 5 min. A paper towel was used to remove excess water or oil from boiled or fried potatoes, respectively. 

All samples (raw and cooked) were frozen at −60 °C for 24 h, freeze-dried (CoolSafe PRO, Labogene, Denmark) and ground. Such homogenized powder of raw, boiled, and fried FCP was analyzed for phenolics and sugars, while acrylamide content was determined only in fried samples. 

### 2.6. Phenolics Analysis

#### 2.6.1. Extraction of Phenolics

Extraction of phenolics was conducted as previously described by Dite Hunjek et al. [28]. Briefly, phenolics from homogenized freeze-dried samples (0.5 g) were extracted with 5 mL of 80% methanol with 1% formic acid (*v*/*v*), using ultrasonic bath (Elmasonic 40H, Elma, Germany) for 30 min at 50 °C. After centrifugation at 3000 rpm/10 min (Hettich^®^ Rotofix 32a, Tuttlingen, Germany), the procedure was repeated by adding 5 mL of extraction solvent to precipitate. Supernatants were combined into a 10 mL flask and made up with solvent. Extracts were filtered (0.45 µm membrane filter, Macherey-Nagel GmbH & Co. KG, Düren, Germany) into vials and stored at −20 °C until the UPLC MS^2^ analysis. Extractions were performed in a duplicate (*n* = 2).

#### 2.6.2. UPLC MS^2^ Analysis of Phenolics

UPLC MS^2^ analysis was performed using an Agilent series 1290 RRLC instrument linked to a triple quadrupole mass spectrometer (6430) (Agilent Technologies, Santa Clara, CA, USA)) with an ESI ion source. Zorbax Eclipse Plus C18 column (100 × 2.1 mm, 1.8 µm) (Agilent Technologies) was used for the separation. Chromatographic conditions, as well as instrument settings, solvent composition, and gradient conditions were as previously described by Elez Garofulić et al. [29]. Briefly, column temperature was set at 35 °C, the injection volume was 2.5 µL, and flow rate 0.3 mL min^−1^. The eluent A was 0.1% of formic acid (*v*/*v*) and eluent B 0.1% formic acid in acetonitrile (*v*/*v*). Ionization was performed by electrospray (ESI) in negative and positive mode (*m*/*z* 100–1000). Data were collected in the dynamic multiple reaction monitoring mode (dMRM). The retention time and mass spectra of the phenolic standards were used to identify compounds, while quantification was performed using calibration curves obtained from the standards. Analytical parameters for chlorogenic acid standard were: six concentrations in a range of 1.625–30 mg L^−1^, regression equation: y = 3639.4x + 575.69, R^2^ = 0.9999, LOD = 0.453 mg L^−1^, and LOQ = 1.371 mg L^−1^. Results are expressed as mg 100 g^−1^ of dry weight (DW). Dry weight was determined by drying lyophilized potato samples at 103 ± 1 °C (FN-500, Nüve, Ankara, Turkey) to constant weight (AOAC, 1990) [30].

### 2.7. Sugar Analyses

#### 2.7.1. Extraction of Sugars

The extraction of sugars was performed according to the method described by Dite Hunjek et al. [28]. The 4 mL of 80% methanol (*v*/*v*) was added into 0.4 g of the ground freeze-dried sample. The mixture was homogenized using vortex and thermostated in a water bath at 60 °C for 60 min. After centrifugation at 6000 rpm/15 min supernatant was filtered and collected into a 5 mL flask and made up with solvent. Extracts were filtered through 0.45 µm membrane filter into vials and stored at +4 °C until the analyses. Extractions were performed in a duplicate (*n* = 2).

#### 2.7.2. HPLC Analysis of Sugars

HPLC analysis of sugars (fructose, glucose, and sucrose) was performed using an Agilent 1260 Infinity quaternary LC system (Agilent Technologies) equipped with refractive index detector (RID). Compounds were separated on a Cosmosil Sugar-D, 5 µm, 250 × 4.6 mm I.D. (Nacalai Tesque, INC., Kyoto, Japan) column. The chromatographic conditions were as described by Dite Hunjek et al. [28]: mobile phase (80% acetonitrile, *v*/*v*) was in isocratic elution mode at flow rate of 1.3 mL min^−1^, injection volume was 10 µL, and the column temperature was set at 45 °C. Identification and quantification of sugars was conducted by comparing retention times and peak areas with the one obtained from standard solutions. Standard solutions were prepared in 80% ethanol *(v*/*v*) and a fixed concentration of each sugar standard was used for quantification. The results are expressed as g 100 g^−1^ DW.

### 2.8. Acrylamide Analysis

#### 2.8.1. Extraction of Acrylamide

In order to determine the content of acrylamide in fried FCP, the method given by Al-Taher [31] was applied with some modifications and without using internal standard [24]. Freeze-dried fried samples (1 g) were homogenized on a vortex with 5 mL of *n*-hexane, after which 10 mL of water and 10 mL of acetonitrile were added and vortexed for 3 min. In such prepared mixture QueChERS salt packet was added and it was strongly shaken for 1 min. After centrifugation at 5000 rpm/5 min, the hexane layer was discarded and 1 mL from acetonitrile layer was transferred into 2 mL vial packed with QueChERS d-SPE salts. Mixture was homogenized at vortex and centrifuged at 5000 rpm/1 min. Supernatant (0.5 mL) was transferred into vials and analyzed by UPLC MS^2^. Extractions were performed in a duplicate (*n* = 2).

#### 2.8.2. UPLC MS^2^ Analysis of Acrylamide

The UPLC MS^2^ analysis of acrylamide was performed as previously described by Dite Hunjek et al. [28] by Agilent UPLC system (Section 2.6.2). A Hypercarb TM column (5 µm, 50 mm × 2.1 mm) with a guard column (5 µm, 10 mm × 2 mm) (Thermo Hypersil-Keystone, Bellefonte, PA, USA) was used for the separation, column temperature was set at 22 °C, injection volume was 10 µL, and flow rate 0.7 mL min^−1^. Mobile phase was 10% methanol with 0.1% formic acid. Ionization was done by electrospray (ESI) in positive ion mode. The identification of acrylamide was confirmed by comparing the peak ratios of MRM transitions *m*/*z* 72 → 55.1 from sample extracts and standard solutions. Quantification was performed using a calibration curve from extracted acrylamide standard solutions. Analytical parameters for acrylamide standard were: six concentrations in a range of 20–500 ng mL^−1^, regression equation: y = 55.042x − 124.76, R^2^ = 0.9999, LOD = 7.479 ng mL^−1^, and LOQ = 22.666 ng mL^−1^. The results are expressed as µg kg^−1^ DW.

### 2.9. Statistical Analysis

The statistical analysis was carried out to examine the influence of the UV-C treatment (0, 3, 5, and 10 min), storage time (0, 8, 11, 15, and 23 days) and cooking method (raw, boiled, and fried FCP) on the content of chlorogenic acid, fructose, glucose, and sucrose as well as on the content of acrylamide in fried FCP. The experimental data were analyzed using Statistica ver. 12.0 software (Statsoft Inc., Tulsa, OK, USA). Data were tested for normality by the Shapiro–Wilk test, while homoscedasticity was tested by Levene’s test. All dependent variables were examined using ANOVA (parametric data) or Kruskal–Wallis test (nonparametric data). Means within groups were compared with Tukey’s HSD test or Kruskal–Wallis test. The statistically obtained results are shown in Table 1 and Table 2 as mean values ± standard error (SE). SE is expressed as the standard deviation of the sampling distribution for all analyzed samples which were taken in statistical processing and calculated by the above-mentioned software. The grand mean represents the mean of all results obtained for a particular chemical component. The relationships between compounds (chlorogenic acid and reducing sugars in raw FCP and acrylamide in fried FCP) were tested by calculated Spearman’s rank correlation coefficients. For Principal Component Analysis (PCA) XLSTAT ver. 2020.5.1 software (Addisoft, Paris, France) was applied. PCA was based on a correlation matrix of samples using values of chlorogenic acid, fructose, glucose, and sucrose in order to examine the possible grouping of the samples by the UV-C treatment, storage time, and cooking method. Analysis involved principal components (PC) with eigenvalue > 1 and variables with communalities ≥ 0.5. The significance level for all tests was *p* ≤ 0.05. 

## 3. Results and Discussion

### 3.1. Phenolics Analysis

According to the obtained results of phenolics, chlorogenic acid was the predominant compound in all FCP samples, while caffeic acid and rutin were present in concentrations below LOQ values, while *p*-coumaric acid and catechin were not detected. Therefore, only the results of chlorogenic acid are given in Table 1. Previously, other authors also found chlorogenic acid with its isomers as the most abundant phenolic compound (90%) in tubers [32]. It is located mostly in potato peel, followed by outer flesh. Catechin, rutin, and *p*-coumaric acid are also mainly located in potato peel but, depending on variety, they can be found in outer and/or inner flesh of potato tuber [32]. Generally, the phenolics content in potatoes is determined by various factors, such as cultivar type, growing and harvesting conditions, climatic conditions, crop maturity, and storage conditions [33,34,35].

Grand mean (GM) value of chlorogenic acid content was 9.67 mg 100 g^−1^ DW, which is slightly higher when compared to the chlorogenic acid GM of the same potato variety in the 2017 harvest (7.46 mg 100 g^−1^ DW) [28]. Statistical analysis did not show a significant effect of UV-C treatment on the content of chlorogenic acid, although a slight decrease with increasing of UV-C dose can be observed. This trend was additionally proved by the interaction of the cooking method vs. UV-C treatment, which distinguished the raw unirradiated control samples from the raw UV-C treated ones. The content of chlorogenic acid was significantly lower in UV-C treated samples, and it decreased with higher dose applied. It could be suggested that under the conditions of the present experiment, irradiation of FCP could have increased the activity of PPO, but more likely it could have decreased the activity of the PAL. In support of this, noticeable browning was not observed in irradiated samples during the experiment. The decrease of total phenolics due to increased UV-C dose was also reported for irradiated fresh-cut spinach [14]. On the contrary, Teoh et al. [7] noticed an increase of total phenolics in FCP which were irradiated before packaging and stored at +4 °C in permeable plastic boxes. This disagreement with the results of the present study could be, among other things, the result of various experimental conditions. However, when observing the influence UV-C treatment 75.53% of the initial amount of chlorogenic acid was retained after 10-UV-C, and 88.34% after 3-UV-C. Higher retention of chlorogenic acid is favorable since it has diverse health benefits, such as preventing cancer, cardiovascular diseases, and diabetes as well as anti-inflammatory effects [33,36,37]. In addition, chlorogenic acid from potatoes is found to produce an increase in insulin sensitivity and a decrease in gut glucose absorption as well as it prevents gluconeogenesis [33,38]. However, in terms of the browning process, lower content of chlorogenic acid is preferable [39].

No significant changes in the content of chlorogenic acid were observed during storage of FCP. The interaction of UV-C treatment vs. storage days did not show significant differences in the amount of chlorogenic acid between the FCP samples, although numerically, control had slightly higher values in comparison with UV-C treated samples during the entire storage period. However, the interaction of cooking method vs. storage days gives a better insight of the influence of storage time on the content of chlorogenic acid in raw FCP. Its levels decreased during the whole storage period, still retaining 78.99% at the 23rd day. Other authors also observed a decrease in phenolics during storage of FCP [7] which could be explained by the participation of chlorogenic acid as a substrate for PPO in oxidation reactions leading to tissue browning [40]. 

Boiling and frying significantly reduced the levels of chlorogenic acid when compared to raw samples. The same occurrence was observed by Tudela et al. [41]. In their study all cooked (boiled, steam boiled, fried, and microwaved) FCP samples of cv. Mona Lisa had a significantly lower content of caffeic acid derivates (50% of their initial amount). Additionally, the same authors reported higher values of caffeic acid derivates in boiled potatoes in comparison with fried ones. Contrary, Blessington et al. [42] observed higher levels of phenolics in cooked potato samples as opposed to raw samples, while the lowest content of phenolics was found in boiled potatoes compared to baked, fried, and microwaved ones. Although the statistical analysis in the present study did not show the difference between boiled and fried samples with respect to chlorogenic acid amounts, the loss of chlorogenic acid was more pronounced in boiled (55.5%) than in fried (53.6%) FCP. Reduction in phenolics in cooked FCP could be associated to cell degradation which occurs during cooking at high temperatures, thus resulting in more easily release of phenolics and their further dissolution in water [43]. Furthermore, the content of phenolics in cooked potatoes depends not only on the method and conditions of cooking [42,43,44], but as well on the other factors such as oxidative enzyme action, solubility, interconversion of compounds, etc. [34]. Despite the decrease of chlorogenic acid amounts in raw FCP during storage, its content in cooked FCP was fairly uniform regardless of storage day (interaction cooking method vs. storage days). The interaction of cooking method vs. UV-C treatment did not show a clear effect of irradiation on the content of chlorogenic acid in the cooked samples. 

### 3.2. Sugars Analysis

As presented in Table 1, glucose (GM 0.292 g 100 g^−1^ DW) and sucrose (GM 0.281 g 100 g^−1^ DW) were the most abundant sugars, followed by fructose (GM 0.212 g 100 g^−1^ DW). The content of reducing sugars was higher, while the amount of sucrose was lower than it was observed by Dite Hunjek et al. [28] who reported GM of glucose 0.17 g 100 g^−1^ DW, GM of fructose 0.13 g 100 g^−1^ DW, and GM of sucrose 0.54 g 100 g^−1^ DW in FCP of the same cultivar harvested in 2017. The differences in levels of sugars were probably caused by different treatment, pre- and post-harvest factors, such as specific climatic conditions during growth [45,46].

UV-C treatment significantly affected fructose and sucrose, in particular, 5-UV-C treatment significantly increased its amount. Their values were approximately 1.5- to 2.0-fold higher when compared to their values in control or 3-UV-C treated samples. The same trend was observed for glucose (1.4-fold higher), although it was not significant. The UV-C radiation can affect the activity of several enzymes related to sugar metabolism, and therefore alter sugars amount in fruits and vegetables [15,47]. It could be assumed that UV-C irradiation, primarily 5-UV-C applied on FCP, also affected enzyme activity resulting in sugars increase. Therefore, for better understanding of the effect of UV-C irradiation on FCP and consequently on the content of sugars, additional studies should be performed. Still, in comparison with other FCP samples, higher amounts of sugars in 5-UV-C treated samples were clearly noticeable throughout the entire storage period except at the first day (UV-C treatment vs. storage days). It can also be observed that the levels of sugars in 3-UV-C and 10-UV-C irradiated FCP did not differ significantly from the control. These results indicate the importance of selecting the most adequate irradiation dose that would result in a lower increase of sugars, since it is associated with a lower potential of acrylamide forming during frying [48].

According to the statistical analysis, fructose content significantly increased during storage and it was even more pronounced after the 15th day. Although it was statistically insignificant, the content of glucose and sucrose increased as well, while sucrose content decreased after the 15th day. These changes during refrigerated storage could be the result of a low-temperature sweetening [15,49], as well as a consequence of sucrose hydrolysis by enzyme invertase. Similar increase in glucose and fructose amounts was observed in the last days of storage in UV-C treated peaches [47]. 

Regardless of the cooking method, the sugar content decreased and difference between boiled and fried samples was almost indistinguishable. This reduction in boiled FCP samples was probably due to solubility of sugars in water and sugars leaking [50,51] and in fried samples reducing sugars can undergo Maillard reaction to form color and aroma compounds and, to a lesser extent, acrylamide by reaction with L-asparagine [20,52,53]. Literature data showed variability in the content of sugars in cooked potatoes according to the cooking method and conditions [15,54]. Observing the interaction of cooking method vs. UV-C treatment for the raw samples, it can be noticed that all analyzed sugars were present in the highest amounts in raw 5-UV-C irradiated samples. Despite this significant excess of sugars in raw 5-UV-C samples, further cooking lowered it remarkably and there were no significant differences in levels of sugars between the samples after cooking. Regardless of the storage day, raw FCP was characterized with significantly higher content of sugars (cooking method vs. storage days), while there was no particular trend for cooked FCP samples.

### 3.3. Acrylamide Analysis

UV-C treatment of raw FCP had a significant influence on the content of acrylamide in fried FCP, while the influence of raw FCP storage on the acrylamide amounts was not observed (Table 2). GM of acrylamide content was 597.47 µg kg^−1^ DW. 

It was observed that all samples treated with UV-C were described with higher acrylamide content in comparison with control, however, 5-UV-C irradiated raw samples had the highest acrylamide content of 762.47 µg kg^−1^ DW what was approx. 1.5-fold higher when compared to control and 3-UV-C treated samples. Here it should be noted that the highest content of reducing sugars (0.96 g 100 g^−1^ DW) was also found in raw 5-UV-C treated samples. Since reducing sugars are precursors in Maillard’s reactions, their increased content could have a significant role in acrylamide formation [55]. Accordingly, calculated Spearman’s correlation coefficient showed a strong positive correlation between the content of acrylamide in fried and reducing sugars in raw samples (r = 0.74), while moderate positive correlation (r = 0.53) characterized the relation of acrylamide and sucrose amounts. This is in line with previously reported observations [28,55]. The proposed level of reducing sugars in potato tubers intended for roasting or frying is below 1 g kg^−1^ FW and it can ensure a formation of acrylamide below 500 μg kg^−1^ FW [56]. As it can be seen, regardless of the applied UV-C treatment the content of sugars (Table 1) and acrylamide (Table 2) was always below the assumed limits which according to ECR 2017/2158 for acrylamide is 750 μg kg ^−1^ FW, noting that the values expressed as DW are about three-fold higher than those expressed as FW. Similar increase in acrylamide level was observed by Sobol et al. [18] in fried potatoes produced from irradiated potato tubers. 

Storage of raw FCP did not significantly affect the acrylamide amounts in fried FCP, although a numerical increase was observed, reaching the highest level on the 23rd day (669.77 µg kg^−1^ DW). The results of interaction UV-C treatment vs. storage days confirmed the significant impact of UV-C treatment where the highest levels of acrylamide were detected in 5-UV-C sample regardless of the day of storage. However, all obtained acrylamide values were below the assumed limits. Furthermore, there was a strong negative correlation (r = −0.77) between the content of acrylamide in fried FCP and chlorogenic acid in raw samples, which is in accordance with Zhu et al. [57] and Kalita et al. [58] who also found a negative correlation between the amounts of acrylamide and total phenolics as well as acrylamide and chlorogenic acid. 

### 3.4. PCA Analysis

**Figure 1:**
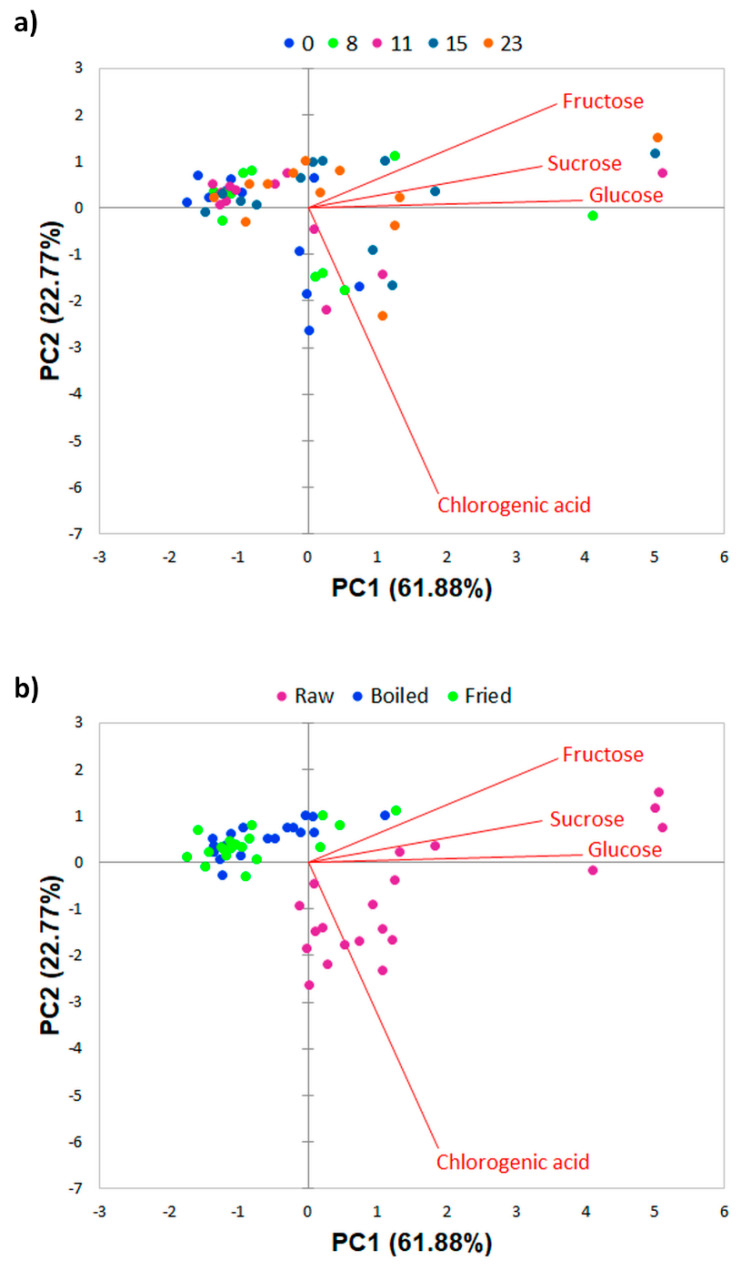
Distribution of UV-C untreated and treated fresh-cut potatoes in two-dimensional coordinate system defined by the first two principal components (PC1 and PC2) in relation to the (**a**) storage days and (**b**) cooking method.

**Figure 2:**
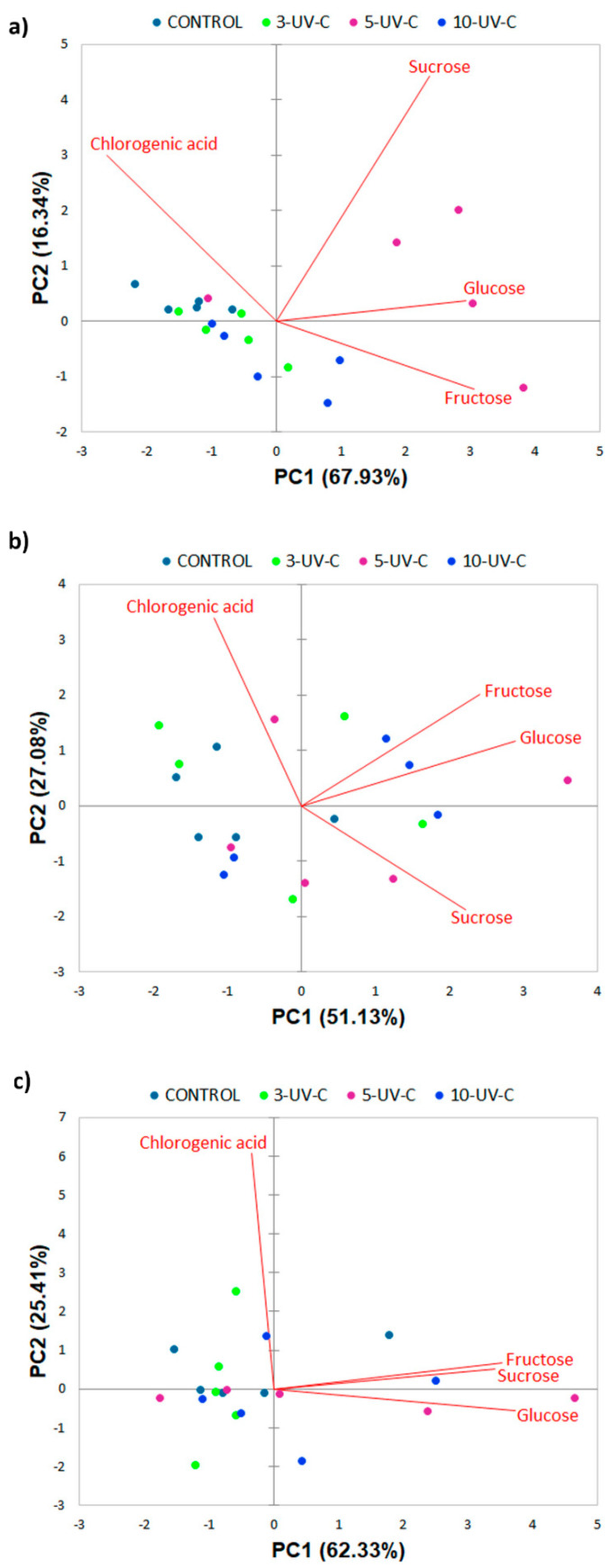
Distribution of (**a**) raw, (**b**) boiled, and (**c**) fried UV-C untreated and treated fresh-cut potatoes in two-dimensional coordinate system defined by the first two principal components (PC1 and PC2).

In addition to ANOVA, PCA was also performed in order to test a possible separation of the FCP samples by the content of chlorogenic acid and individual sugars with respect to UV-C treatment, storage days, and cooking method. Obtained biplots of the distribution of FCP samples in relation to the storage time and method of cooking are presented in Figure 1, while the biplots of the distribution of raw, boiled, and fried FCP according to the applied UV-C treatment are given in Figure 2.

In terms of storage days and cooking method, PC1 and PC2 together described 84.65% of total variance. Strong positive correlation was present between PC1 and fructose (r = 0.86), glucose (r = 0.95), and sucrose (r = 0.80), while chlorogenic acid was in moderate positive correlation (r = 0.45) with this PC. On the other hand, a very strong negative correlation (r = −0.89) described the relation of chlorogenic acid and PC2. As it can be seen in Figure 1a, FCP samples did not distinguished by the days of storage, which confirms their fairly uniform composition over 23 days. Considering the effect of cooking method, a clear separation of raw FCP samples from the cooked ones is visible (Figure 1b). Raw samples were mostly at positive PC1 values and were described with higher amounts of chlorogenic acid and sugars, unlike the majority of boiled and fried samples grouped mainly at negative PC1 values. 

Regarding influence of UV-C treatment on raw FCP samples, PC1 and PC2 explained 87.74% of total variance (Figure 2a). PC1 was in negative strong correlation with chlorogenic acid (r = −0.78), while positive strong/very strong correlation was present between this PC and fructose (r = 0.91), glucose (r = 0.88), and sucrose (r = 0.71). PC2 showed positive moderate correlation only with sucrose (r = 0.65). Biplot showed separation of 5-UV-C treated samples, which were located mainly at positive PC1 values being characterized with higher levels of sugars. This is in accordance with previously discussed results (Table 1). 

As for boiled FCP, total variation of the analytical parameters was 78.21% (Figure 2b) PC1 was in strong positive correlation with fructose (r = 0.76) and sucrose (r = 0.70) and in very strong correlation with glucose (r = 0.91), while PC2 was in strong positive correlation with chlorogenic acid (r = −0.78). As it can be seen, the majority of the control samples were grouped at negative values of PC1, having a lower content of sugars. According to the effect of UV-C irradiation on fried samples, PC1 and PC2 together described 87.74% of the variance (Figure 2c). Correlation between PC1 and fructose, glucose, and sucrose was positive and very strong (r = 0.90, 0.95 and 0.87, respectively), while positive very strong correlation described the relation between PC2 and chlorogenic acid (r = 0.99). A certain grouping of control and 3-UVC samples is noticeable at negative values of PC1, being characterized with lower levels of sugars when compared to 5-UV-C and 10-UV-C samples. These results are in line with already discussed observations given by interaction cooking method vs. UVC treatment (Table 1). 

## 4. Conclusions

The obtained results revealed that applied UV-C treatments caused the slight reduction of chlorogenic acid and the increase of total sugars in the raw FCP samples, but still in acceptable concentrations. The observed increase in sugars, which was particularly pronounced when 5-UV-C irradiation was applied, did not affect the acrylamide safety of the product, as all fried samples contained acrylamide levels below the limit value approved by EFSA and EU Commission Regulation 2017/2158. Furthermore, FCP samples maintained a relatively stable chemical composition during 23 days of storage. The results of this study showed that UV-C irradiation could certainly be of interest to the fresh-cut industry along with the well-known germicidal action, but further studies are needed.

## Figures and Tables

**Table 1:** The influence of UV-C treatment, storage days, and cooking method on chlorogenic acid (mg 100 g^−1^ DW) and sugars (g 100 g^−1^ DW) in fresh-cut potato.

Source of Variation	Chlorogenic Acid	Fructose	Glucose	Sucrose
UV-C treatment	*p* = 0.052	*p* = 0.005 *	*p* = 0.078	*p* < 0.001 *
Control	11.32 ± 1.06 ^a^	0.179 ± 0.006 ^a^	0.259 ± 0.015 ^a^	0.212 ± 0.012 ^a^
3-UV-C	10.00 ± 0.80 ^a^	0.191 ± 0.012 ^a^	0.259 ± 0.012 ^a^	0.226 ± 0.011 ^a^
5-UV-C	8.82 ± 0.66 ^a^	0.267 ± 0.022 ^b^	0.364 ± 0.035 ^a^	0.435 ± 0.058 ^b^
10-UV-C	8.55 ± 0.57 ^a^	0.210 ± 0.014 ^ab^	0.286 ± 0.013 ^a^	0.253 ± 0.014 ^ab^
Storage days	*p* = 0.5968	*p* < 0.001 *	*p* = 0.06	*p* = 0.069
0	9.98 ± 1.14 ^a^	0.150 ± 0.005 ^a^	0.238 ± 0.011 ^a^	0.257 ± 0.013 ^a^
8	9.65 ± 0.95 ^a^	0.177 ± 0.011 ^ab^	0.296 ± 0.024 ^a^	0.307 ± 0.040 ^a^
11	9.57 ± 0.90 ^a^	0.196 ± 0.011 ^bc^	0.276 ± 0.022 ^a^	0.307 ± 0.063 ^a^
15	9.58 ± 0.77 ^a^	0.260 ± 0.021 ^c^	0.309 ± 0.023 ^a^	0.323 ± 0.037 ^a^
23	9.59 ± 0.81 ^a^	0.275 ± 0.020 ^c^	0.341 ± 0.035 ^a^	0.214 ± 0.017 ^a^
Cooking method	*p* < 0.001 *	*p* = 0.001 *	*p* < 0.001 *	*p* < 0.001 *
Raw	15.20 ± 0.52 ^b^	0.260 ± 0.018 ^b^	0.397 ± 0.023 ^b^	0.393 ± 0.044 ^b^
Boiled	6.77 ± 0.14 ^a^	0.194 ± 0.009 ^a^	0.244 ± 0.009 ^a^	0.231 ± 0.014 ^a^
Fried	7.05 ± 0.19 ^a^	0.181 ± 0.008 ^a^	0.235 ± 0.011 ^a^	0.220 ± 0.012 ^a^
UV-C treatment x storage days	*p* = 0.360	*p* = 0.766	*p* = 0.603	*p* = 0.293
Control × 0	11.47 ± 3.00 ^a^	0.152 ± 0.003 ^a^	0.232 ± 0.009 ^a^	0.228 ± 0.010 ^a^
3-UV-C × 0	9.16 ± 2.60 ^a^	0.149 ± 0.006 ^a^	0.228 ± 0.017 ^a^	0.227 ± 0.020 ^a^
5-UV-C × 0	10.44 ± 2.31 ^a^	0.147 ± 0.016 ^a^	0.227 ± 0.031 ^a^	0.266 ± 0.030 ^a^
10-UV-C × 0	8.83 ± 1.33 ^a^	0.154 ± 0.014 ^a^	0.266 ± 0.027 ^a^	0.305 ± 0.030 ^a^
	*p* = 0.324	*p* = 0.003 *	*p* = 0.240	*p* = 0.020 *
Control × 8	10.48 ± 2.29 ^a^	0.165 ± 0.008 ^ab^	0.234 ± 0.029 ^a^	0.235 ± 0.030 ^ab^
3-UV-C × 8	10.36 ± 1.74 ^a^	0.138 ± 0.010 ^a^	0.269 ± 0.019 ^a^	0.207 ± 0.017 ^a^
5-UV-C × 8	8.79 ± 1.81 ^a^	0.253 ± 0.022 ^b^	0.417 ± 0.072 ^a^	0.525 ± 0.125 ^b^
10-UV-C × 8	8.98 ± 2.10 ^a^	0.154 ± 0.010 ^ab^	0.265 ± 0.021 ^a^	0.261 ± 0.015 ^ab^
	*p* = 0.176	*p* = 0.051	*p* = 0.416	*p* = 0.294
Control × 11	11.13 ± 2.64 ^a^	0.191 ± 0.006 ^a^	0.247 ± 0.019 ^a^	0.202 ± 0.034 ^a^
3-UV-C × 11	10.69 ± 1.84 ^a^	0.169 ± 0.009 ^a^	0.272 ± 0.047 ^a^	0.226 ± 0.030 ^a^
5-UV-C × 11	8.54 ± 1.30 ^a^	0.250 ± 0.036 ^a^	0.345 ± 0.064 ^a^	0.586 ± 0.223 ^a^
10-UV-C × 11	7.91 ± 1.16 ^a^	0.174 ± 0.011 ^a^	0.240 ± 0.028 ^a^	0.213 ± 0.009 ^a^
	*p* = 0.415	*p* = 0.015 *	*p* = 0.120	*p* = 0.020 *
Control × 15	11.38 ± 2.04 ^a^	0.177 ± 0.017 ^a^	0.270 ± 0.052 ^a^	0.2140 ± 0.020 ^a^
3-UV-C × 15	9.54 ± 1.89 ^a^	0.241 ± 0.025 ^ab^	0.252 ± 0.019 ^a^	0.265 ± 0.031 ^ab^
5-UV-C × 15	8.40 ± 1.21 ^a^	0.360 ± 0.053 ^b^	0.407 ± 0.053 ^a^	0.530 ± 0.097 ^b^
10-UV-C × 15	8.98 ± 0.82 ^a^	0.261 ± 0.032 ^ab^	0.305 ± 0.029 ^a^	0.281 ± 0.051 ^ab^
	*p* = 0.329	*p* = 0.104	*p* = 0.528	*p* = 0.575
Control × 23	12.12 ± 2.65 ^a^	0.210 ± 0.016 ^a^	0.313 ± 0.047 ^a^	0.179 ± 0.032 ^a^
3-UV-C × 23	10.27 ± 1.22 ^a^	0.260 ± 0.032 ^a^	0.274 ± 0.030 ^a^	0.206 ± 0.024 ^a^
5-UV-C × 23	7.91 ± 0.52 ^a^	0.324 ± 0.065 ^a^	0.423 ± 0.130 ^a^	0.267 ± 0.052 ^a^
10-UV-C × 23	8.06 ± 0.95 ^a^	0.306 ± 0.018 ^a^	0.353 ± 0.025 ^a^	0.203 ± 0.019 ^a^
Cooking method × storage days	*p* < 0.001 *	*p* = 0.094	*p* = 0.001 *	*p* = 0.003 *
Raw × 0	17.28 ± 1.07 ^b^	0.159 ± 0.008 ^a^	0.278 ± 0.011 ^b^	0.308 ± 0.020 ^b^
Boiled × 0	6.32 ± 0.20 ^a^	0.158 ± 0.010 ^a^	0.248 ± 0.020 ^b^	0.256 ± 0.020 ^ab^
Fried × 0	6.33 ± 0.35 ^a^	0.134 ± 0.008 ^a^	0.189 ± 0.011 ^a^	0.206 ± 0.012 ^a^
	*p* < 0.001 *	*p* = 0.235	*p* = 0.001 *	*p* = 0.009 *
Raw × 8	15.83 ± 0.50 ^b^	0.198 ± 0.021 ^a^	0.388 ± 0.050 ^b^	0.448 ± 0.100 ^b^
Boiled × 8	6.93 ± 0.50 ^a^	0.149 ± 0.010 ^a^	0.205 ± 0.012 ^a^	0.220 ± 0.010 ^a^
Fried × 8	6.20 ± 0.30 ^a^	0.185 ± 0.023 ^a^	0.296 ± 0.029 ^ab^	0.253 ± 0.040 ^a^
	*p* < 0.001 *	*p* = 0.018 *	*p* < 0.001 *	*p* = 0.027 *
Raw × 11	15.02 ± 1.20 ^b^	0.239 ± 0.028 ^b^	0.395 ± 0.038 ^b^	0.503 ± 0.169 ^b^
Boiled × 11	6.64 ± 0.31 ^a^	0.170 ± 0.007 ^a^	0.228 ± 0.013 ^a^	0.256 ± 0.031 ^ab^
Fried × 11	7.04 ± 0.21 ^a^	0.178 ± 0.007 ^ab^	0.204 ± 0.007 ^a^	0.161 ± 0.025 ^a^
	*p* < 0.001 *	*p* = 0.006 *	*p* < 0.001 *	*p* = 0.031 *
Raw × 15	14.21 ± 0.99 ^b^	0.348 ± 0.044 ^b^	0.414 ± 0.039 ^b^	0.459 ± 0.084 ^b^
Boiled × 15	7.01 ± 0.25 ^a^	0.243 ± 0.016 ^ab^	0.291 ± 0.022 ^a^	0.285 ± 0.043 ^ab^
Fried × 15	7.51 ± 0.40 ^a^	0.188 ± 0.019 ^a^	0.221 ± 0.018 ^a^	0.224 ± 0.022 ^a^
	*p* = 0.001 *	*p* = 0.014 *	*p* = 0.001 *	*p* = 0.002 *
Raw × 23	13.65 ± 1.60 ^b^	0.357 ± 0.042 ^b^	0.512 ± 0.071 ^b^	0.247 ± 0.041 ^b^
Boiled × 23	6.97 ± 0.22 ^a^	0.250 ± 0.019 ^ab^	0.247 ± 0.017 ^a^	0.140 ± 0.008 ^a^
Fried × 23	8.15 ± 0.49 ^a^	0.219 ± 0.021 ^a^	0.264 ± 0.026 ^a^	0.255 ± 0.008 ^b^
Cooking method × UV-C treatment	*p* < 0.001 *	*p* = 0.012 *	*p* = 0.003 *	*p* < 0.001 *
Raw × Control	19.29 ± 0.45 ^c^	0.193 ± 0.009 ^a^	0.343 ± 0.026 ^a^	0.234 ± 0.023 ^a^
Raw × 3-UV-C	15.73 ± 0.43 ^b^	0.225 ± 0.025 ^ab^	0.333 ± 0.018 ^a^	0.278 ± 0.009 ^a^
Raw × 5-UV-C	13.27 ± 0.93 ^a^	0.381 ± 0.044 ^b^	0.575 ± 0.055 ^b^	0.754 ± 0.112 ^b^
Raw × 10-UV-C	12.50 ± 0.57 ^a^	0.242 ± 0.031 ^ab^	0.337 ± 0.018 ^a^	0.306 ± 0.031 ^a^
	*p* = 0.204	*p* = 0.492	*p* = 0.206	*p* = 0.245
Boiled × Control	7.07 ± 0.18 ^a^	0.169 ± 0.007 ^a^	0.210 ± 0.012 ^a^	0.201 ± 0.019 ^a^
Boiled × 3-UV-C	7.09 ± 0.42 ^a^	0.193 ± 0.023 ^a^	0.239 ± 0.009 ^a^	0.210 ± 0.023 ^a^
Boiled × 5-UV-C	6.53 ± 0.25 ^a^	0.209 ± 0.019 ^a^	0.257 ± 0.019 ^a^	0.288 ± 0.037 ^a^
Boiled × 10-UV-C	6.40 ± 0.14 ^a^	0.204 ± 0.019 ^a^	0.270 ± 0.022 ^a^	0.226 ± 0.021 ^a^
	*p* = 0.050 *	*p* = 0.136	*p* = 0.219	*p* = 0.052
Fried × Control	7.59 ± 0.24 ^b^	0.174 ± 0.014 ^a^	0.225 ± 0.017 ^a^	0.200 ± 0.019 ^a^
Fried × 3-UV-C	7.19 ± 0.58 ^ab^	0.156 ± 0.006 ^a^	0.206 ± 0.011 ^a^	0.190 ± 0.009 ^a^
Fried × 5-UV-C	6.64 ± 0.13 ^a^	0.210 ± 0.021 ^a^	0.259 ± 0.030 ^a^	0.262 ± 0.038 ^a^
Fried × 10-UV-C	6.76 ± 0.41 ^ab^	0.184 ± 0.019 ^a^	0.250 ± 0.020 ^a^	0.227 ± 0.008 ^a^
Grand mean	9.67	0.212	0.292	0.281

* Values are significant at *p* ≤ 0.05. Results are expressed as mean ± SE. Different letters within column mean statistically different values at *p* ≤ 0.05.

**Table 2:** The influence of UV-C treatment, storage, and cooking method on acrylamide (µg kg^−1^ DW) in fried fresh-cut potato.

Source of Variation	Acrylamide
UV-C treatment	*p* < 0.001 *
Control	480 ± 17.0 ^a^
3-UV-C	558 ± 10.2 ^a^
5-UV-C	763 ± 26.5 ^b^
10-UV-C	590 ± 21.4 ^ab^
Storage days	*p* = 0.187
0	530 ± 35.6 ^a^
8	573 ± 30.5 ^a^
11	586 ± 47.7 ^a^
15	628 ± 45.1 ^a^
23	670 ± 44.9 ^a^
UV-C treatment × storage days	*p* = 0.003 *
Control × 0	390 ± 15.9 ^a^
3-UV-C × 0	544 ± 25.1 ^bc^
5-UV-C × 0	649 ± 20.5 ^c^
10-UV-C × 0	539 ± 4.8 ^b^
	*p* = 0.006 *
Control × 8	490 ± 11.9 ^a^
3-UV-C × 8	558 ± 7.4 ^a^
5-UV-C × 8	701 ± 28.1 ^b^
10-UV-C × 8	544 ± 23.2 ^a^
	*p* = 0.001 *
Control × 11	481± 15.3 ^a^
3-UV-C × 11	518 ± 15.2 ^a^
5-UV-C × 11	798 ± 29.6 ^b^
10-UV-C × 11	547 ± 7.3 ^a^
	*p* = 0.001 *
Control × 15	502 ± 14.4 ^a^
3-UV-C × 15	572 ± 9.3 ^ab^
5-UV-C × 15	818 ± 32.6 ^c^
10-UV-C × 15	620 ± 10.7 ^b^
	*p* = 0.001 *
Control × 23	537 ± 12.5 ^a^
3-UV-C × 23	597 ± 11.2 ^a^
5-UV-C × 23	847 ± 15.5 ^c^
10-UV-C × 23	699 ± 25.1 ^b^
Grand mean	670

* Values are significant at *p* ≤ 0.05. Results are expressed as mean ± SE. Different letters within column mean statistically different values at *p* ≤ 0.05.
